# Parametric investigation of an injection-jet self-powered Fontan circulation

**DOI:** 10.1038/s41598-022-05985-3

**Published:** 2022-02-09

**Authors:** Ray Prather, Arka Das, Michael Farias, Eduardo Divo, Alain Kassab, William DeCampli

**Affiliations:** 1 Department of Mechanical and Aerospace Engineering, University of Central Florida, 4000 Central Florida Blvd., Orlando, FL 32816 USA; 2 Department of Mechanical Engineering, Embry-Riddle Aeronautical University, 1 Aerospace Blvd., Daytona Beach, FL 32114 USA; 3 The Heart Center, Arnold Palmer Hospital for Children, 92 West Miller Street, Orlando, FL 32806 USA; 4 College of Medicine, University of Central Florida, 6850 Lake Nona Blvd, Orlando, FL 32827 USA

**Keywords:** Interventional cardiology, Translational research

## Abstract

Approximately $1/2500$ babies are born with only one functioning ventricle and the Fontan is the third and, ideally final staged palliative operation for these patients. This altered circulation is prone to failure with survival rates below $50\%$ into adulthood. Chronically elevated inferior vena cava (IVC) pressure is implicated as one cause of the mortality and morbidity in this population. An injection jet shunt (IJS) drawing blood-flow directly from the aortic arch to significantly lower IVC pressure is proposed. A computer-generated 3D model of a 2–4 year old patient with a fenestrated Fontan and a cardiac output of 2.3 L/min was generated. The detailed 3D pulsatile hemodynamics are resolved in a zero-dimensional lumped parameter network tightly-coupled to a 3D computational fluid dynamics model accounting for non-Newtonian blood rheology and resolving turbulence using large eddy simulation. IVC pressure and systemic oxygen saturation were tracked for various IJS-assisted Fontan configurations, altering design parameters such as shunt and fenestration diameters and locations. A baseline “failing” Fontan with a 4 mm fenestration was tuned to have an elevated IVC pressure (+ 17.8 mmHg). Enlargement of the fenestration to 8 mm resulted in a 3 mmHg IVC pressure drop but an unacceptable reduction in systemic oxygen saturation below 80%. Addition of an IJS with a 2 mm nozzle and minor volume load to the ventricle improved the IVC pressure drop to 3.2 mmHg while increasing systemic oxygen saturation above 80%. The salutary effects of the IJS to effectively lower IVC pressure while retaining acceptable levels of oxygen saturation are successfully demonstrated.

## Introduction

### Background

A structurally normal heart consists of two separate ventricles, one pumping de-oxygenated blood returning from the body to the lungs, and the other pumping oxygenated blood from the lungs to the body. Approximately 1/2500 babies are born with only one functioning ventricle^1^. These patients do not survive unless a series of staged palliative operations are performed to assure adequate flow to both the pulmonary and systemic circulation. In general, the single ventricle (SV) palliation is completed in stages, typically designated as stage I, II and III. Physiologically, at Stage 1 the vasculature is reconstructed to assure unobstructed and roughly equal flow to both the lungs (“pulmonary circulation”) and body (“systemic circulation”), both circulations powered “in parallel” by the ventricle. At this stage, the ventricle’s volume load is roughly twice normal, increasing the strain on the myocardium, and systemic oxygen delivery is below normal because oxygenated blood mixes with deoxygenated blood in the ventricle. At stage II the pulmonary circulation is decoupled from the ventricle. The superior vena cava is connected directly to the pulmonary arteries (“cavopulmonary connection” or “bidirectional Glenn”) to supply pulmonary blood flow passively, i.e., without a pump. Although systemic oxygen content is still below normal, the excess volume load on the ventricle is eliminated. At Stage III, referred to as the *Fontan procedure*^2^, the inferior vena cava (IVC) is decoupled from the heart and its flow routed to the pulmonary arteries typically through a synthetic conduit (“Fontan conduit”) placed outside the heart. Systemic oxygen content is now closer to normal with the majority of venous return going through the lungs rather than returning to the systemic circulation. This is typically called the “Fontan circulation”. Ideally, as long as the impedance of the lung vasculature is sufficiently low, the entire circulatory blood volume travels “passively” through the lungs without the assistance of a pumping ventricle. The driving force becomes the transpulmonary pressure gradient (the difference between vena cava pressure, or “Fontan pressure”, and atrial pressure). Even with normal pulmonary vascular resistance (PVR), vena cava pressure (P_*fontan*_) must rise from its usual value of 3–6 mmHg to values of 10–14 mmHg.

A substantial proportion of patients with the Fontan circulation do not do well. A study of 683 adult Fontan patients published in 2018 from the Australian and New Zealand Fontan Registry found that, by age 40, mortality reached 20%, only 53% of patients were free of heart failure symptoms, and only 41% were free of serious adverse events^1^. Similar data has been widely reported^2–18^. Approximately half of the instances of mortality and significant morbidity can be attributed to the failure of the unique Fontan circulatory system. The physiological associations with “Fontan failure” are complex and the mechanisms incompletely understood.

Fundamentally, taking the pump out of the Fontan circulation renders the pulmonary vasculature resistance (PVR) the “critical bottleneck” for setting the total circulatory volume flow rate (systemic cardiac output, ${Q}_{s}$)^19–22^. This aberration is especially evident during exercise, during which the ventricle pumps harder and faster, but without an appropriate rise in ${Q}_{s}$, compromising exercise capacity^23^. Additionally, an insidious and vicious cycle develops whereby chronically reduced blood return to the ventricle stimulates (1) systemic vasoconstriction, increased ventricular afterload, and systolic dysfunction, and (2) myocardial remodeling which increases ventricular end-diastolic pressure and diastolic dysfunction. The cycle continues as increased end-diastolic pressure reduces the trans-pulmonary pressure gradient and hence the driving force of pulmonary blood flow, further reducing preload and ${Q}_{s}$ unless ${P}_{fontan}$ increases further^23^. As a result of this insidious cycle, or of a pathological increase in PVR, in many failing Fontan patients, ${P}_{fontan}$ may ultimately rises above values of 10–14 mmHg, typical of stable Fontan patients. While the association of elevated ${P}_{fontan}$ with subsequent morbidity is somewhat inconsistent and controversial, it is widely believed that a value exceeding 18 mmHg itself contributes to chronic venous congestion and life-shortening complications such as liver dysfunction, protein losing enteropathy, and plastic bronchitis.

In an attempt to mitigate the problems associated with the Fontan circulation, clinicians have utilized a “fenestration”—a 3–4 mm connection between the Fontan conduit and the atrium—to allow some venous blood to enter the ventricle directly. Other than reducing some short-term adverse effects of the Fontan operation, however, the fenestration does not seem to influence the long-term effects of the Fontan circulation. Investigators have proposed the use of mechanical pumps to assist the circulation that are powered extracorporeally akin to conventional ventricular assist devices. The problems with these devices—thrombosis, stroke, mechanical malfunction and infection—are well-known. The engineering problems are formidable, and a Fontan-specific device has yet to move to clinical trials^24^.

### Proposed solution

Given these problems, in 2017 we proposed an alternative solution. We proposed to utilize the principle of the injection jet, energized by the ventricle itself, to power the Fontan circulation^33^. The jet would be produced using a tapered synthetic graft sewn onto the aorta (or one of its branches) and inserted into the lumen of the Fontan conduit (henceforth referred to as the “IJS”). In principle, the high velocity “nozzle” flow would transfer momentum to the surrounding Fontan circulation flow by the mechanism of entrainment, effectively lowering IVC pressure upstream of the jet^25–31^. This concept was first investigated in a computational multi-scale model for a small number of conditions and geometric configurations in which entrainment was demonstrated, but with only a small decrease in IVC pressure^32–43^.

Unlike other pumps, the IJS is entirely intra-corporeal, with no moving parts, and is powered by the native ventricle. In practice, the IJS jet is driven by the pressure gradient across the shunt, namely the pressure difference between the systemic venous return (14–18 mmHg) and the systemic arterial pressure (40–90 mmHg). Hence, the jet-pump power originates from the cardiovascular system itself, effectively making this a self-powered pump, as opposed to externally powered pumps such as ventricular assist devices with ex-vivo power sources. In some sense, our self-powered IJS approach is in the spirit of mechanical or thermal energy harvesters that avoid external power requirements for implanted biomedical devices^44,45^.

In the present study, several anatomic design modifications are made, and the modeling of the entrainment is substantially refined. The important role of the fenestration and of the nozzle size are emphasized. The critical role of time-dependent turbulence in momentum transfer is demonstrated. Following considerable parameter exploration, it is concluded that the IJS mechanism can indeed decrease ${P}_{fontan}$ by 3–4 mmHg and increase ventricular preload with a modest decrease in arterial oxygen content. The effect was sensitive to the geometric configuration of the IJS, but robust to changes in physiological parameters. These results provided the first computational evidence that this surgically feasible construct can potentially lower ${P}_{fontan}$ while tapping the reserve power of the ventricle at a clinically acceptable ratio of cardiac output (CO) to systemic flow (Qs) (CO/Qs < 1.5).

## Methods

### Geometries

**Figure 1:**
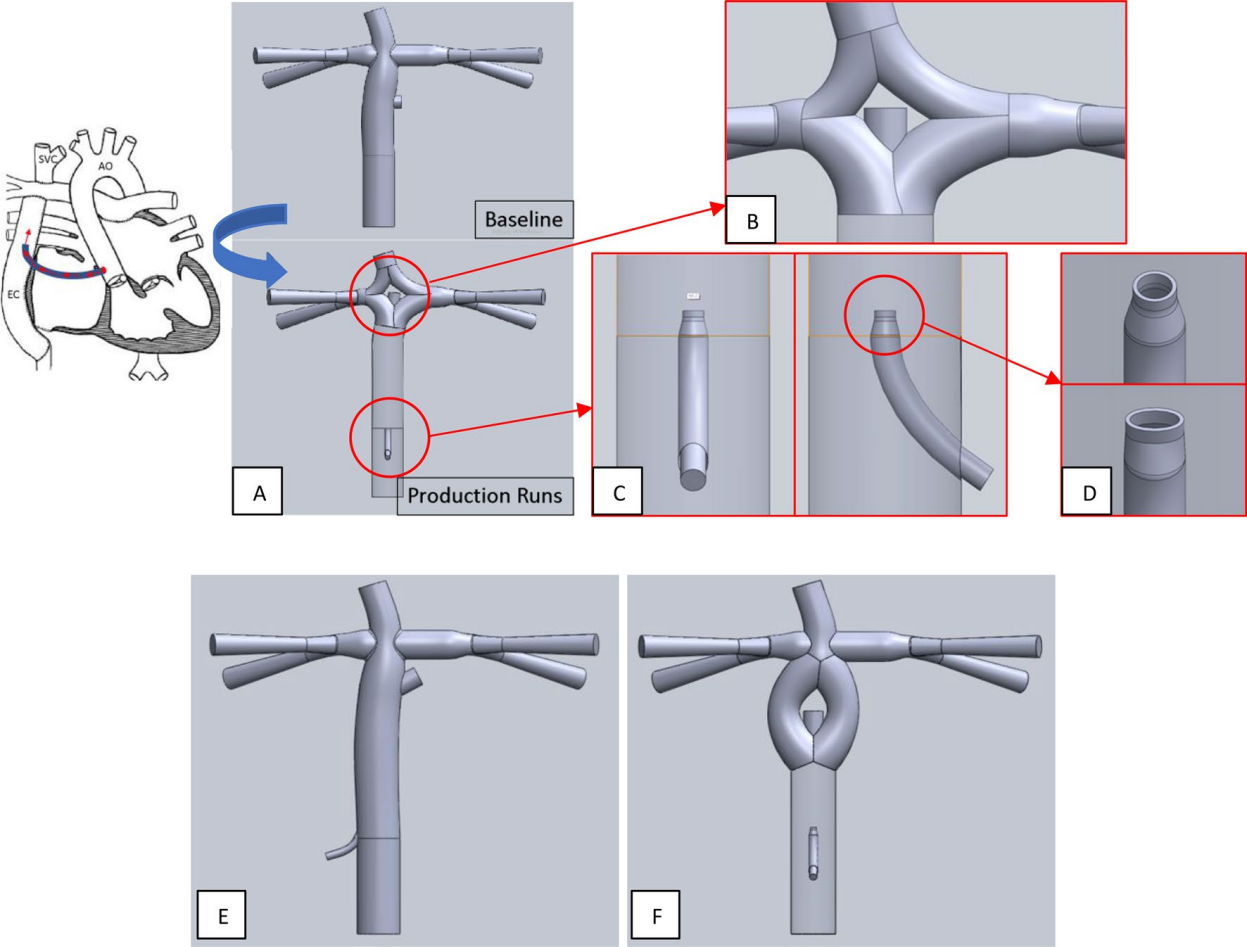
(**A**,**B**) Example geometric alteration of a traditional fenestrated Fontan (baseline) to create a double Y-graft (2Y) connection with the IJS and a fenestration at the bifurcation; (**C**) single nozzle IJS in the Fontan conduit with (**D**) variable nozzle shape (circular (top) and high aspect-ratio elliptical mouth (bottom)). A 2 mm IJS nozzle and a 8 mm fenestration are combined in (**E**) a skew configuration where the jet originates at a shallow angle pointing to a lateral fenestration and (**F**) a torus configuration that retains the centerline-aligned fenestration in the cusp of the bifurcation. Note that the bifurcating connection between the superior vena cava and the pulmonary arteries (as used in the configuration of **B**) is changed to a more conventional, single connection. The latter is more surgically appealing.

Representative geometries were solid-modeled based on measurements gathered from angiograms and catheterization reports of 2–4 year old patients. Body surface area (BSA) of ~ 0.7 m^2^ was used. Figure 1A shows a traditional fenestrated Fontan (the “baseline model” in this study) and, as an example, a fenestrated “double Y-graft (2Y)” configuration with the IJS. This 2Y configuration has been shown to reduce energy loss^46^. All models in this study have the fenestration located at the bifurcation of the Fontan conduit (Fig. 1B). Surgically, the fenestration is either connected directly to the atrium or (more likely) by way of a short graft between the two. The IJS graft enters the side wall of the Fontan conduit, and the nozzle is located at the center of the lumen. The nozzle can take various shapes (Fig. 1C,D).

Multi-scale computational models based on the configurations in Fig. 1B were run to explore the design parameter space to find trends toward progressive decrease in P_*fontan*_. Parameters included IJS position, nozzle shape, nozzle diameter, and fenestration diameter. This was done by “eyeballing” results rather than by using an automated optimization process. Promising parameter values were then implemented in a set of alternative IJS-Fontan configurations that were considered more surgically appealing (Fig. 1E,F) and these configurations were also investigated numerically to determine if they indeed provided the targeted caval pressure drop under appropriate IJS parameter values obtained from the double y-split IJS studies.

### Mesh

As shown in Supplementary Fig. S1-A online the mesh is generated using a polyhedral mesh with 3 prism layers, a maximum size of 0.6 mm, mean target size of 0.6 mm and a minimum of 0.036 mm (with curvature, surface and volume refinements). Volumetric mesh refinement targets increased accuracy in tracking the jet-ambient flow shear layer and the vortex isostructures emerging from viscous shearing (Supplementary Fig. S1-B online). First, refinement occurs extruding from the shunt’s overall surface (6–12% of target size) to the surrounding volume; second, in the wake of the IJS outlet, the region is refined (6–12% of target size) fanning radially at a 7° angle for 3–5 cm downstream. Polyhedral meshes are well suited for complex geometry flow analysis due to their shape which easily adapts to irregular topologies retaining high mesh quality and promoting stability, while tetrahedral elements are likely to generate slivers impacting calculations^47^. Given the increased number of shared faces with neighboring cells, polyhedral cells result in improved gradient calculations. Documented studies observe polyhedral meshes to exhibit better convergence properties requiring ~ 60% less iterations with ~ 77% fewer cells (including a prism layer) compared to tetrahedral meshes^48^. Overall converged meshes result in approximately 1.5 million polyhedral.

### Lumped parameter model (LPM)

The boundary conditions (BC) at all inlets and outlets of the IJS-assisted Fontan required by the computational fluid dynamics (CFD) flow solver are provided by a low-order lumped parameter model (LPM) of the peripheral circulation that is based on multi-degree of freedom Windkessel compartment models previously described^32^. This circuit is tuned to replicate pressure and flow rate waveforms with documented patient data^49–59^ and present the appropriate impedance to the flow solver presented by the peripheral circulation. The SV Fontan circulation is simplified by lumping peripheral vascular beds in several coupled beds that model arterial and venous beds separately. In this case, the lower body is lumped in a single compartment, while the upper body is lumped in two vascular beds for the carotid arteries and the subclavian arteries, and the coronaries are lumped in a single compartment as well. For simplicity and due to low flow inertia, inductors are omitted in all venous compartments. Heart valves are modeled by a resistance in series with a diode and produce unidirectional flow. To drive the system, the ventricle in the LPM is represented by a time-varying capacitor that acts as a pumping heart via the “double hill” normalized elastance function^60^. The resulting system of 32 coupled linear ordinary differential equations for the pressure (voltage) and volume flow rate (current) is solved by an in-house 4th order adaptive Runge–Kutta method.

### Hemodynamics and control

The hemodynamic analysis is carried out with the commercial CFD software StarCCM + (Siemens, Munich, Germany) that provides an integrated meshing and flow solver environment. The flow solver provides the time-resolved pulsatile hemodynamics by numerically solving the conservation of mass (continuity equation) and momentum equations. Blood density is taken as 1060 kg/m^3^ while the viscous stress tensor $\sigma =\mu \left(\dot{\gamma }\right)\left[\nabla V+{\left(\nabla V\right)}^{T}\right]$ accounts for the non-Newtonian rheology of blood through a shear-rate dependent viscosity $\mu \left(\dot{\gamma }\right)$ provided by the Carreau-Yasuda model. Hematocrit of 40% is assumed^61^. A two-phase flow analysis is conducted by assuming a N-phase mixture using local averaged flow quantities to resolve the flow field. Although blood flows as a single-phase fluid, the purpose of the two-phase model is to enable precise tracking of the IJS flow as it interacts with the Fontan conduit co-flow. This is important in the design of the IJS and in proper oxygen tracking. The domain walls of the solid model are assumed to be rigid.

In a typical Fontan circulation, the low ambient flow velocities ($\sim 25\frac{cm}{s}$) corresponds to a Reynolds number of $Re\approx 1200$. However, the high-speed IJS jet in peak systole exhibits speeds up to 17 times larger than the ambient flow (~ 425 cm/s) leading to jet Reynolds numbers ranging between $Re$ ≈ 2120 to 3385 which is well in the turbulent regime. Moreover, while at peak systole, the IJS jet flow can be considered turbulent, in diastole the same IJS jet flow becomes laminar with speed as low as 200–250 cm/s ($Re\approx 1100$). To capture this transition from laminar to turbulent regimes and to capture the complex shear layer interactions between the background IVC and IJS jet co-flows, a Large Eddy Simulation (LES) solver with a WALE (Wall-Adapted Local Eddy-viscosity) sub-grid model is adopted. LES is well-suited for complex flow interactions of the shear layer present at the interface of the IVC co-flow and the pulsatile jet produced by the IJS and has the additional benefit of being able to track laminar-to-turbulent and turbulent-to-laminar transitions as well as fine details of the shear layer structures of the unsteady jet-entrainment that are key to modeling the effective IJS pumping action responsible for the IVC pressure drop.

A second-order implicit scheme with a multistep predictor–corrector backwards differential formulation (BDF) for stable and accurate time-resolved solutions is used in the CFD solver. The time-step is set to 0.333–3.33 ms (200–2000 time-steps over the heart cycle) which combined with the converged mesh average base-size meets the Courant number stability and time-accuracy requirements (Co < 1). Convection was modeled using a bounded central differencing scheme which combines the benefits of a central and second-order upwind schemes mediated by a blending function. Time-step convergence criteria for continuity, x-momentum, y-momentum, and z-momentum was set at 10^–3^, and time-step inner (sublevel) iterations were limited to 50. A mesh independence study has been conducted for all sample geometries to ensure grid independent solutions.

The boundary conditions at all the inlets (SVC, IVC, and IJS) are set as mass flow rates, while the boundary conditions at the outlets (RPA, LPA and fenestration) are set as pressures. The values of these boundary conditions are provided by the interaction with the LPM at each time step-level^32,35^.

The IJS produces a left-to-right shunt that induces an excess load on the ventricle. Multi-scale models that resulted in CO/Q_s_ > 1.5 were not considered acceptable on the grounds that this condition would possibly result in long term deleterious effects on the myocardium and clinical congestive heart failure. In the presence of a small to moderate left-to-right shunt (CO/Qs < 1.5), systemic flow (Q_s_) would be ideally maintained in the long term by the modulation of SVR and adjustment of stroke volume. To model this, a controller was designed to automatically manipulate SVR and the elastance function. A proportional gain controller maintained systemic flow between 2.1 and 2.3 L/min by modulating ventricular preload and SVR to mimic the ideal physiological response.

### Oxygen tracking

The presence of a fenestration may decrease systemic oxygen content (${C}_{aO2}$), depending on its size and its interaction with the IJS flow. Thus, oxygen tracking is critical to determining optimal IJS-assisted Fontan models^62–64^. In order to determine the degree of ${O}_{2}$ rich IJS flow mixing with low ${O}_{2}$ IVC flow, two-phase flow modeling is carried out to determine the volume fraction (${VF}_{ijs}$) of IJS flow absorbed by the fenestration. This volume fraction estimation is used to accurately determine the systemic ${O}_{2}$ saturation reflecting this anatomy.

**Figure 2:**
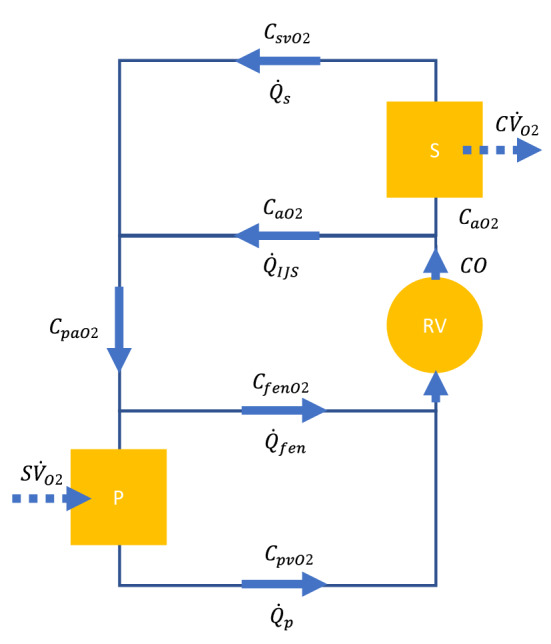
Generic oxygen transport model schematic ($\mathrm{CO}=\mathrm{cardiac output }$[mL/min], ${\mathrm{Q}}_{\mathrm{ijs}}=\mathrm{IJS flow }$[mL/min], ${\mathrm{Q}}_{\mathrm{fen}}=\mathrm{fenestration flow }$[mL/min], ${\mathrm{Q}}_{\mathrm{s}}=\mathrm{systemic flow }$[mL/min], ${\mathrm{Q}}_{\mathrm{p}}=\mathrm{pulmonary flow }$[mL/min$]$, ${\mathrm{C}\dot{\mathrm{V}}}_{\mathrm{O}2}=9\cdot\;\mathrm{m }$[mL/min] oxygen consumption/uptake with $\mathrm{m}=\mathrm{patient weight }[\mathrm{kg}]$, ${\mathrm{C}}_{\mathrm{xxO}2}=\mathrm{oxygen concentration}$ for *a* arterial, *sv* systemic venous, *pa* pulmonary arterial, *pv* pulmonary venous, *S* systemic circulation, and *P* pulmonary circulation).

**Figure 3:**
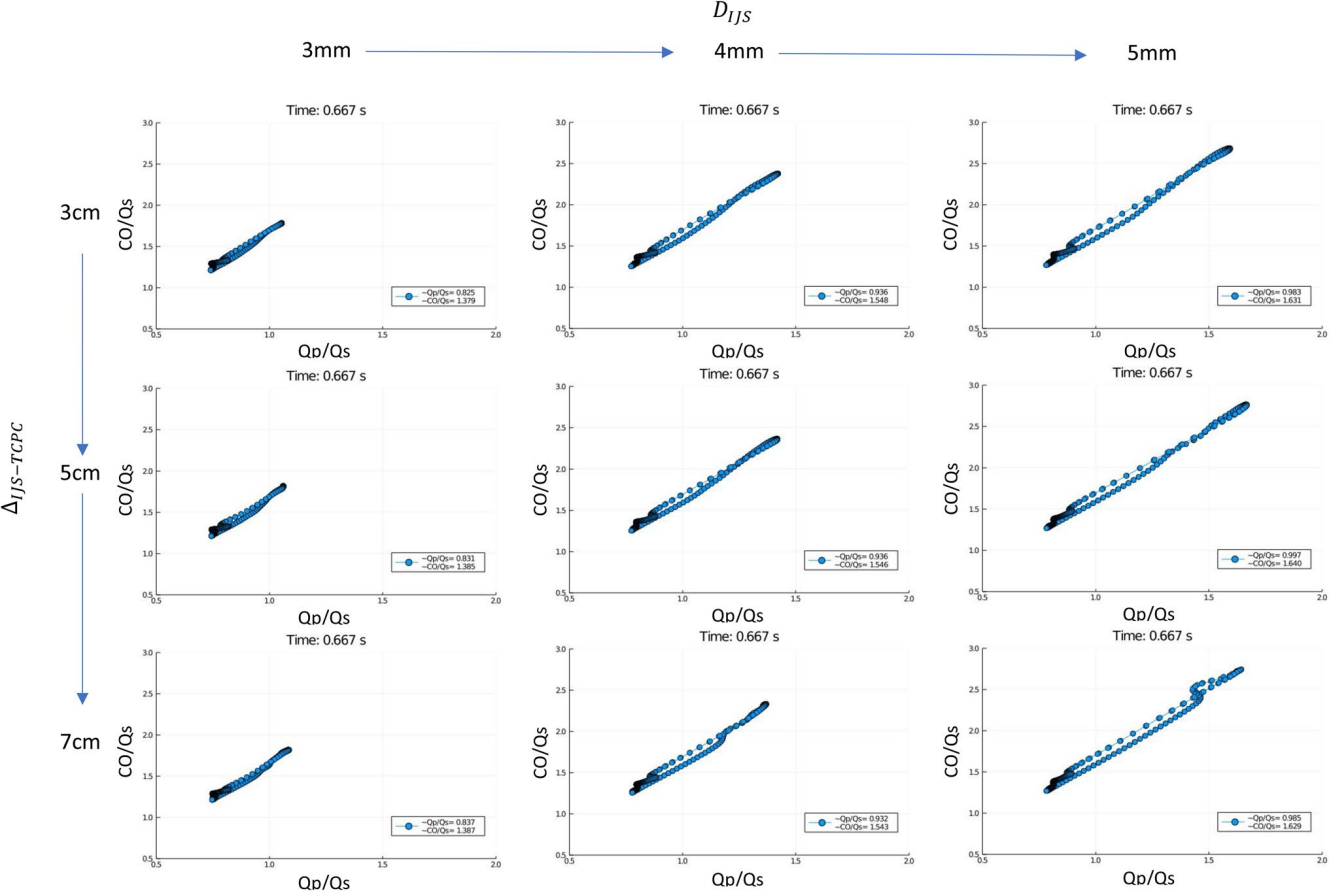
Hysteresis loops for preliminary parameter search from cases 3 to 11 in Table 1 for constant fenestration diameter and varying IJS diameter and IJS-TCPC distance (time-averaged Qp/Qs and CO/Qs are reported in the legend in each subplot).

The schematic in Fig. 2 represents a 1D oxygen transport model for a generalized Fontan circulation that includes an IJS and a fenestration. Steady-state conditions are assumed, hence O_2_ intake by the lungs is exactly balanced by systemic O_2_ consumption: $S{\dot{V}}_{O2}=C{\dot{V}}_{O2}$ and based on Fig. 3, a set of 4 equilibrium equations can be derived (Eqs. 1–4). All flow rates are CFD-derived surface-averaged and time-averaged quantities, while the systemic and venous oxygen concentrations are being calculated.

1 $${C}_{aO2}{\dot{Q}}_{s}-C{\dot{V}}_{O2}={C}_{svO2}{\dot{Q}}_{s}$$

2 $${C}_{paO2}{\dot{Q}}_{p}+S{\dot{V}}_{O2}={C}_{pvO2}{\dot{Q}}_{p}$$

3 $${C}_{svO2}{\dot{Q}}_{s}+{C}_{aO2}{\dot{Q}}_{IJS}={C}_{paO2}{\dot{Q}}_{p}+{C}_{fenO2}{\dot{Q}}_{fen}$$

4 $${C}_{aO2}CO={C}_{fenO2}{\dot{Q}}_{fen}+{C}_{pvO2}{\dot{Q}}_{p}$$

Equations (1–2) describe the systemic and pulmonary oxygen equilibrium, whereas Eqs. (3–4) address shunting balances. Following some manipulation, an expression for ${C}_{aO2}$ can be derived given converged CFD output ($CO$, ${Q}_{s}$, ${Q}_{p}$, ${Q}_{IJS}$, ${Q}_{fen}$ and ${VF}_{ijs}$) and physiologically assumed quantities such as pulmonary venous saturation ${C}_{pvO2}$ and oxygen consumption rate ${C\dot{V}}_{O2}$. The pulmonary O_2_ concentration, ${C}_{pvO2}$, can be derived from ${C}_{pvO2}={O2sat}_{p}\cdot \gamma$ where ${O2sat}_{p}$ is the pulmonary saturation (values of 95–100% are used in this study) and $\gamma [\frac{mL O2}{mL blood}]$ the blood oxygen capacity.

5 $${C}_{aO2}{\dot{Q}}_{s}={C}_{pvO2}CO\frac{1}{1+\frac{{\dot{Q}}_{ijs}}{{\dot{Q}}_{s}}}-{C\dot{V}}_{O2}\frac{\left(1-{VF}_{ijs}\right){\dot{Q}}_{fen}}{{\dot{Q}}_{p}}$$

Equation (5) is used to track systemic oxygen saturation. This general model can also be reduced to a fenestrated Fontan (${\dot{Q}}_{ijs}=0$, $CO={\dot{Q}}_{s}$) and a non-fenestrated Fontan (${\dot{Q}}_{fen}=0$, $CO={\dot{Q}}_{s}$) and could be altered to account for systemic-to-pulmonary arterial collaterals.

### Coupling

To properly capture the momentum transfer, the updates between the CFD and LPM must occur at every time step. This is referred to as “tight coupling”^32,65–68^. The area of interest is removed from the LPM and replaced with the CFD model shown in Fig. 1A. The LPM is now open and completely dependent upon the CFD. In this scheme, the CFD leads the LPM in time and the boundary conditions are relaxed from the CFD to the LPM^32^. In addition, the CFD-LPM coupling has to be converged at every time-step. To achieve this, the “freeze time” option in StarCCM + helps keep the solution at the current time step while still allowing the boundary conditions to change. In the early cycles, rather than taking the full values from the CFD, these are damped based on the previous cycle values. This “boundary condition relaxation” is calculated by linearly interpolating between the previous value and the new calculated value. This damping is slowly removed after every complete heart cycle until the LPM and CFD have converged to a stable solution. Convergence is measured using normalized least-squares on the LPM-CFD boundary values^32^.

When coupling LPM and CFD models: (1) when passing flow rates (Dirichlet) to the LPM, the interface should be connected by a capacitor and (2) when passing pressures (Neumann) to the LPM, the interface should be connected by an inductor^32,65–68^ to increase algorithm stability.

As the IJS is implemented, it is critical to maintain physiological conditions. This is accomplished via a control algorithm that maintains systemic flow constant by dynamically modulating ventricular preload and SVR to mimic circulatory response in the presence of significant shunting IJS flow within the clinical constraint of augmented CO to nominal Qs ratio of less than 1.5 (CO/Qs < 1.5).

### Ethics declaration

This study does not involve human or animal subjects.

### Approval for animal experiments

This study does not involve human subjects.

### Approval for human experiments

This study does not involve human subjects.

### Consent to participate/Consent to publish

This study does not involve human subjects.

## Results

Models demonstrating a 3.2 mmHg IVC pressure drop with a systemic oxygen saturation greater than 80%, while retaining a constant PVR, were discovered. Three surgical IJS-assisted Fontan anatomic variants were considered, each of which produced a clinically significant IVC pressure drop (models illustrated in Fig. 1B,E,F).

### Baseline

Table 1 shows results from models without the presence of the IJS. The baseline model (Fig. 1A) and the double-Y-graft model (Fig. 1B) with a 4 mm fenestration were tuned to an IVC pressure representing a “failing” Fontan (17.8 mmHg). The fenestration diameter was then enlarged to 7 mm and the two models recalculated. Enlargement of the fenestration to 7 mm itself results in a 3 mm reduction of P_*fontan*_, but sysO2 also decreases significantly to 80–81%. Note also that, for a given fenestration size, the time-averaged quantities (static pressure, flow rates and flow ratios) for the double Y-graft variant (bifurcations of both IVC and SVC connections to the pulmonary arteries, Fig. 1B) did not differ significantly from those of the baseline model.

**Table 1:** Summary table of baseline (4 mm fenestration), enlarged fenestration (7 mm fenestration) and without IJS implementation (Model nomenclature: [TCPC topology] − [fenestration diameter]).

Case #	Model	Qp [L/min]	Qs [L/min]	CO [L/min]	Qp/Qs	CO/Qs	Pivc [mmHg]	Psvc [mmHg]	Plpa [mmHg]	Prpa [mmHg]	IJS VF	sysO_2_ [%] ${C}_{pv{O}_{2}}=100\%$	sysO_2_ [%]${C}_{pv{O}_{2}}=95\%$
1a	4mmfen	2.049	2.268	2.268	0.904	1.000	17.827	17.792	17.012	17.008	–	97	92
2a	2Y-4mmfen	2.057	2.274	2.274	0.905	1.000	17.742	17.780	17.058	17.073	–	97	92
1b	7mmfen	1.305	2.431	2.431	0.537	1.000	14.693	14.662	14.221	14.234	–	80	75
2b	2Y-7mmfen	1.326	2.426	2.426	0.546	1.000	14.701	14.741	14.547	14.354	–	81	76

Pulmonary venous saturations range ${C}_{pv{O}_{2}}=$ {95–100}%.

Transiently, the pulse pressure in this passive circulation is small (< 3 mmHg), making the time-dependence of the pressure waveforms for a fenestrated Fontan less important than that of the flow waveforms. Supplementary Fig. S2 online shows the pulmonary flow rate calculated from the summation of LPA and RPA flow measurements at the CFD domain outlets. It can be observed that for both fenestration sizes the double Y-grafts variant resulted in a mild *Qp* improvement, presumably due to reduced energy loss and better flow organization. The significant drop in *Qp* with an increase in fenestration size is also evident. Clinically, a $Qp/Qs$ for a fenestrated Fontan is expected to range from 0.7 to 0.9 ensuring adequate systemic oxygen saturation. Indeed, $Qp/Qs$ below 0.7 (~ 0.54 in Table 1, case #1b, 2b) is consistent with an unacceptable O_2_ saturation at or below 80%. These models were generated to demonstrate that enlargement of the fenestration alone results in unacceptable systemic oxygen content. Later, it is shown, however, that a fenestration diameter > 4 mm is likely necessary for effective IJS momentum transfer. On the other hand, the IJS can *increase* oxygen saturations by increasing pulmonary flow and also by sending some of its (partially oxygenated) blood through the fenestration.

### Design space exploration

After establishing a set of baseline runs, the IJS was implemented first in the double-Y-graft anatomic variant and geometrical parameters such as IJS size and placement as well as fenestration size were varied. The fenestration was moved in the cusps of the IVC bifurcation and the IJS nozzle is placed through the side wall of the Fontan conduit. The data in Table 2 summarize the parameter sweep results for pressure, flow rate, and systemic oxygen in 3 groups: baseline (described in “Baseline”), group 1 and group 2. Overall, it can be observed that for all cases, the controller designed to maintain *Qs* successfully kept systemic flow within 10% of the baseline cases.

**Table 2:** Summary table of exploration of IJS and fenestration size and placement, including modified double Y-graft (2Y) anatomic variant (model nomenclature: [anatomic variant] − [distance from IJS nozzle to cavopulmonary connection] − [IJS diameter] − [fenestration diameter]).

Case #	Model	Qp [L/min]	Qs [L/min]	CO [L/min]	Qp/Qs	CO/Qs	Pivc [mmHg]	Psvc [mmHg]	Plpa [mmHg]	Prpa [mmHg]	IJS VF	_sysO2_ [%] ${C}_{pv{O}_{2}}=100\%$	sysO_2_ [%] ${C}_{pv{O}_{2}}=95\%$	
1a	4mmfen	2.049	2.268	2.268	0.904	1.000	17.827	17.792	17.012	17.008	–	97	92	Baseline
2a	2Y-4mmfen	2.057	2.274	2.274	0.905	1.000	17.742	17.780	17.058	17.073	–	97	92	
1b	7mmfen	1.305	2.431	2.431	0.537	1.000	14.693	14.662	14.221	14.234	–	80	75	
2b	2Y-7mmfen	1.326	2.426	2.426	0.546	1.000	14.701	14.741	14.547	14.354	–	81	76	
3	2Y-7cmivc-5mmijscircular-6mmfen	2.077	2.243	3.443	0.926	1.535	17.879	18.624	17.834	17.605	0.440	91	86	Group 1
4	2Y-7cmivc-4mmijscircular-6mmfen	2.186	2.431	3.601	0.899	1.481	18.556	19.825	18.787	18.448	0.394	91	86	
5	2Y-7cmivc-3mmijscircular-6mmfen	2.010	2.441	3.324	0.837	1.387	17.339	18.716	17.463	17.118	0.304	90	85	
6	2Y-5cmivc-5mmijscircular-6mmfen	2.065	2.236	3.435	0.924	1.536	17.928	18.755	17.875	17.602	0.432	91	86	
7	2Y-5cmivc-4mmijscircular-6mmfen	2.061	2.301	3.403	0.896	1.479	17.719	18.767	17.751	17.419	0.376	90	85	
8	2Y-5cmivc-3mmijscircular-6mmfen	2.018	2.447	3.329	0.825	1.361	17.312	18.496	17.275	17.183	0.269	89	84	
9	2Y-3cmivc-5mmijscircular-6mmfen	2.104	2.245	3.443	0.937	1.534	18.011	18.770	17.735	17.665	0.380	90	85	
10	2Y-3cmivc-4mmijscircular-6mmfen	2.058	2.297	3.398	0.896	1.479	17.797	18.756	17.670	17.439	0.339	90	85	
11	2Y-3cmivc-3mmijscircular-6mmfen	1.968	2.400	3.296	0.834	1.398	17.341	18.067	17.027	17.295	0.232	87	82	
12	2Y-7cmivc-3mmijscircular-7mmfen	1.629	2.117	2.980	0.769	1.408	15.548	16.830	15.931	15.676	0.316	85	80	Group 2
13	2Y-7cmivc-3mmijscircular-8mmfen	1.756	2.503	3.385	0.701	1.353	15.976	17.387	16.379	16.054	0.286	85	80	
14	2Y-7cmivc-2mmijscircular-6mmfen	1.564	2.224	2.609	0.703	1.173	15.740	15.945	15.408	15.694	0.130	85	80	
15	2Y-7cmivc-2mmijscircular-7mmfen	1.405	2.213	2.639	0.635	1.193	14.905	15.186	14.684	14.972	0.125	81	76	
16	2Y-7cmivc-2mmijscircular-8mmfen	1.387	2.290	2.709	0.606	1.183	14.697	14.950	14.455	14.664	0.119	79	74	

In group 1 (cases 3–11), an initial parametric exploration was carried out by varying only 2 geometric properties, IJS distance to the TCPC ${\Delta }_{IJS-TCPC}$ (3–7 cm) and the IJS nozzle diameter ${D}_{ijs}$ (3–5 mm), while the fenestration diameter was kept constant at 6 mm. This approach revealed the model sensitivity to these parameters as well as a trend in IVC pressure reduction. When comparing ${\Delta }_{IJS-TCPC}$ for constant ${D}_{ijs}$ (cases^3,4,6,7,9,10^ or^5,8,11^), it can be seen that as ${\Delta }_{IJS-TCPC}$ decreases (7 cm to 3 cm) IVC pressure increases. Conversely, comparison for varying ${D}_{ijs}$ for constant ${\Delta }_{IJS-TCPC}$ (cases^3–8^, or^9–11^) shows that for decreasing ${D}_{ijs}$ (5 mm to 3 mm) IVC pressure decreases. Given the values in Table 2, it can be determined that IVC pressure variation due to ${D}_{ijs}$ reduction ranges between 3.1 and 3.8% whereas for a decrease in ${\Delta }_{IJS-TCPC}$, IVC pressure changes 0.24–0.97%. This outcome suggests the model to be more sensitive to shunt diameter than to IJS distance to the TCPC. In the previous section, it was highlighted how incrementing the fenestration size led to a large pressure drop of 3.1 mmHg at the cost of the systemic oxygen saturation falling below 80%. As shown in Table 2, by implementing the IJS, systemic saturations can be significantly improved to over 85%. It can be seen in Table 2 that *Qp* and $Qp/Qs$ recover as well. Upon implementing the IJS, CO/Qs increases, reflecting a significant increase in CO in line with the expected preservation of *Qs* and augmented ventricular preload. Table 1 suggests that the clinical threshold of $CO/Qs\le 1.5$ can only be respected for IJS nozzle diameters of up to 4 mm, as a 5 mm IJS can result in a $CO/Qs>1.6$.

In group 2, the parameter exploration conducted in group 1 is combined with the observations made from the baseline runs: the fenestration diameter is incremented and the IJS diameter is reduced to prevent a volume build-up and increase jet speed. Comparing case 5 with 12–13 for constant ${\Delta }_{IJS-TCPC}$ (7 cm) and ${D}_{ijs}$ (3 mm) and increasing fenestration diameter (7–8 mm), it can inferred that IVC pressure drops by as much as 1.8 mmHg, an expected outcome discussed in “Baseline” (baseline cases). This pressure drop occurs while CO/Qs remains elevated (> 1.3) compared to baseline (1.0). In conjunction with the pressure drop, Qp/Qs (and Qp) decreases from ~ 0.9 to ~ 0.7 which directly leads to a reduction in systemic oxygen saturations as low as 85%. Conversely, in case 14, fenestration diameter (6 mm) and ${\Delta }_{IJS-TCPC}$ are kept constant with an IJS of 2 mm. This results in a IVC pressure drop of 1.6 mmHg, a reduction in CO/Qs to 1.17 and maximum systemic saturation of 85%. Qp/Qs does remain at 0.7. Clearly reducing IJS diameter and incrementing fenestration diameter can be beneficial. Hence in cases 15–16 the implementation of a 2 mm IJS nozzle with an enlarged fenestration (7–8 mm) not only preserves the IVC pressure drop of ~ 3.2 mmHg from the baseline but it improves systemic oxygen saturation with only a small additional volume load to the ventricle ($CO/Qs\approx 1.2$) (compared to cases 1b and 2b). Case 16 presents the largest pressure drop with sustainable saturation achieved so far, this outcome is due to the combined effect of the IJS and the fenestration whose specific configuration allows for entrainment to occur (as shown in the next section), saturations to be maintained, and IVC pressure to be reduced.

### Alternate Fontan conduit configurations

Once the parameters yielding the largest IVC pressure drop in groups 1 and 2 were identified, additional Fontan anatomic variants were investigated (Fig. 1E,F). These models are classified as Group 3.

The significant pressure drop recorded in Table 2 for a 2 mm IJS and an 8 mm fenestration was maintained in cases 17 and 18, with similar systemic oxygen saturations (Table 3). The torus configuration performs best in terms of IVC pressure drop (3.0 mmHg) as opposed to the skew model (2.7 mmHg), but not by much. On the other hand, the skew model shows better systemic oxygen saturation (81%) compared to the torus model (75%). As the parameters used are based on case 16, the reduced Qp/Qs previously observed is retained in both models. The physiological constraint of CO/Q_s_ < 1.5 was maintained. These results encourage further exploration of these alternative surgical configurations.

**Table 3:** Summary table of best IJS and fenestration size and placement implemented in modified Fontan conduit topology (Model nomenclature: [anatomic variant] − [distance between IJS nozzle and cavopulmonary connection] − [IJS diameter] – [fenestration diameter]).

Case #	Model	Qp [L/min]	Qs [L/min]	CO [L/min]	Qp/Qs	CO/Qs	Pivc [mmHg]	Psvc [mmHg]	Plpa [mmHg]	Prpa [mmHg]	IJS VF	sysO_2_ [%] ${C}_{pv{O}_{2}}=100\%$	sysO_2_ [%]${C}_{pv{O}_{2}}=95\%$	
1a	4mmfen	2.049	2.268	2.268	0.904	1.000	17.827	17.792	17.012	17.008	–	97	92	Baseline
2a	2Y-4mmfen	2.057	2.274	2.274	0.905	1.000	17.742	17.780	17.058	17.073	–	97	92	
1b	7mmfen	1.305	2.431	2.431	0.537	1.000	14.693	14.662	14.221	14.234	–	80	75	
2b	2Y-7mmfen	1.326	2.426	2.426	0.546	1.000	14.701	14.741	14.547	14.354	–	81	76	
17	Skew-7cmivc-2mmcircularijs-8mmfen	1.362	2.290	2.719	0.595	1.187	15.081	15.055	14.726	14.669	0.222	81	76	Group 3
18	Torus-3cmivc-2mmcircularijs-8mmfen	1.308	2.301	2.684	0.568	1.166	14.811	14.295	14.795	14.303	0.105	75	71	

### IJS-Fenestration dynamic interaction: the flow ratio hysteresis loop

This group’s previous work^55–65^ focused on time-averaged quantifications (over a heart cycle). Generally, this would be acceptable as the bottom line of this research effort mainly targets a mean IVC pressure drop. However, this model considers a region that combines a native slow (< 50 cm/s), weakly pulsatile (< 3 mmHg pulse pressure) Fontan conduit flow with a high speed (> 350 cm/s), strongly pulsatile IJS flow (> 30 mmHg). As mentioned in “Mesh”, the coupled effects of the IJS and fenestration are the key to the effectiveness of the procedure, as both contribute to IVC pressure reduction. The IJS-fenestration system is highly transient. Simply reviewing average Qp/Qs and CO/Qs in Tables 1 and 2, reveals nothing about the dynamic IJS-fenestration interaction, hence, to visualize IJS loading and fenestration unloading across a cycle, Qp/Qs and CO/Qs are plotted as a transient hysteresis loop in Fig. 3. These plots clearly display the highly dynamic behavior of this model. This tool helps identify potential inefficiencies in the configuration and determine possible solutions.

The hysteresis loops in Fig. 3 help troubleshoot and determine the parameter sensitivity of the model. This figure, obtained from simulations in phase 1, shows how the model is strongly sensitive to IJS diameter and weakly sensitive to IJS-TCPC distance. As IJS diameter is incremented, the loop grows longer, indicating transient fluid accumulation in the Fontan for an expected increase in cardiac output. This is confirmed by the time-averaged quantifications in the legend of each subplot. For increasing IJS diameter (3–5 mm) the Qp/Qs increment ranges between 17.7 and 20.0%, whereas for increasing ${\Delta }_{IJS-TCPC}$, the change is less than 2%. Transiently, as the IJS grows larger, the amount of diverted flow increases, driving the CO higher, hence CO/Qs would equivalently be augmented. In a similar fashion, for a constant fenestration size, as ${D}_{ijs}$ increases, so does Qp/Qs, which is shown to periodically exceed 1.0 by a significant margin. While this occurs only during systole (typically representing $\frac{1}{3}$ of a cardiac cycle), fluid accumulation can still occur if distal resistance, namely PVR, remains constant. This outcome combined with the earlier observation on CO/Qs > 1.5 for a 5 mm shunt, leads to consideration of implementing larger fenestration diameters and smaller IJS nozzle diameters. The added benefit of smaller IJS nozzle diameters would be high jet speeds, potentially leading to higher momentum transfer.

### Flow field

The principal aim of this study is to reduce IVC pressure by implementing an injection jet shunt (IJS) diverting flow from the aorta to the Fontan circulation. A jet can transfer momentum to surrounding flow by means of entrainment. An increase in local momentum can result in a reduction in static pressure. Entrainment induced by a jet pump is the outcome of strong shearing with the co-flowing fluid resulting in eddies and vortices. As aforementioned, the jet flow is found to periodically transition from laminar in diastole to turbulent in systole.

**Figure 4:**
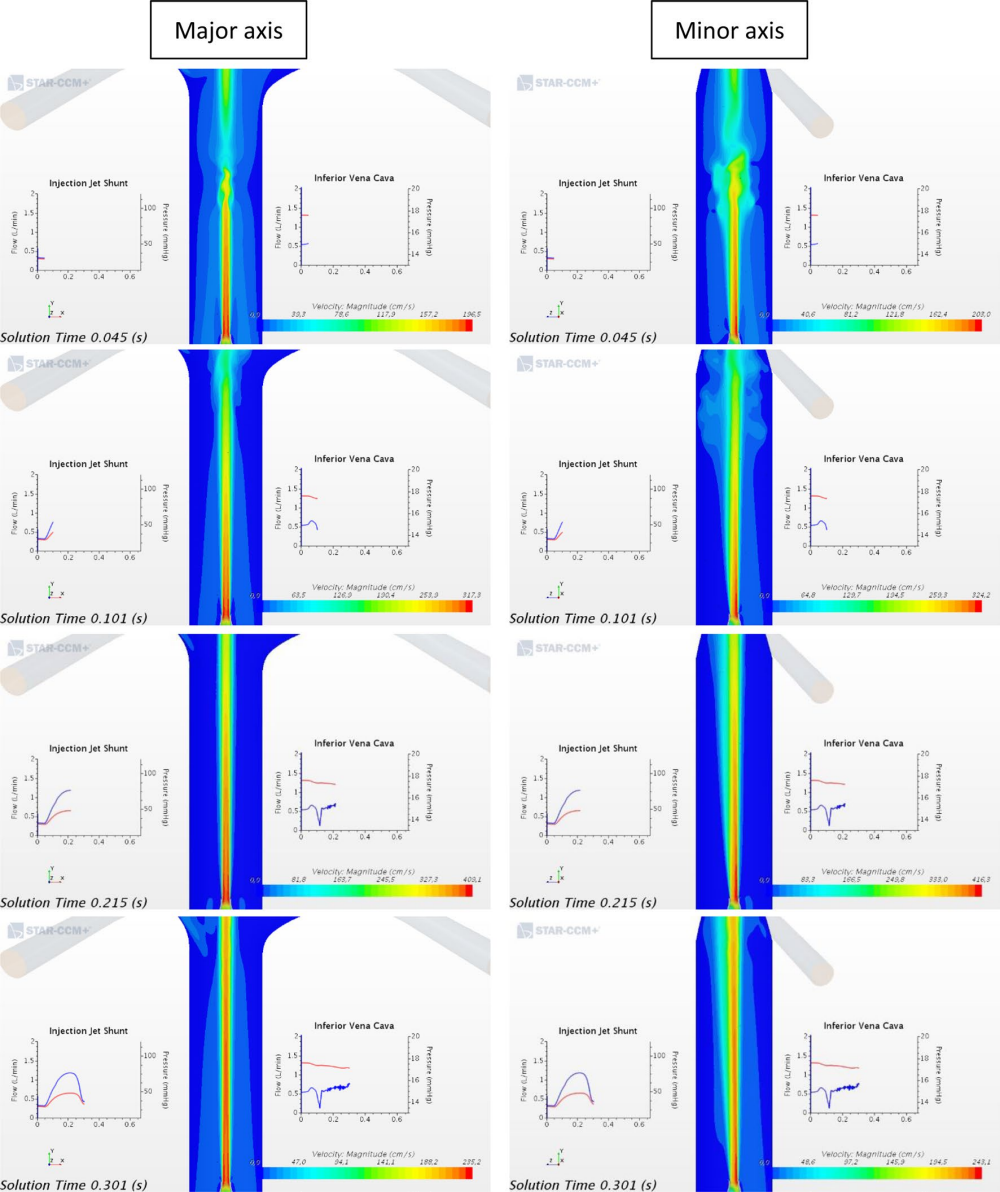
Velocity magnitude contour plots for a 2 mm circular IJS nozzle, 7 cm IJS-TCPC distance and 7 mm fenestration along major and minor axis across a single heart cycle. The x–y plots display pressure (blue) and volume flow rate (red) sampled in the IJS shunt and the IVC.

**Figure 5:**
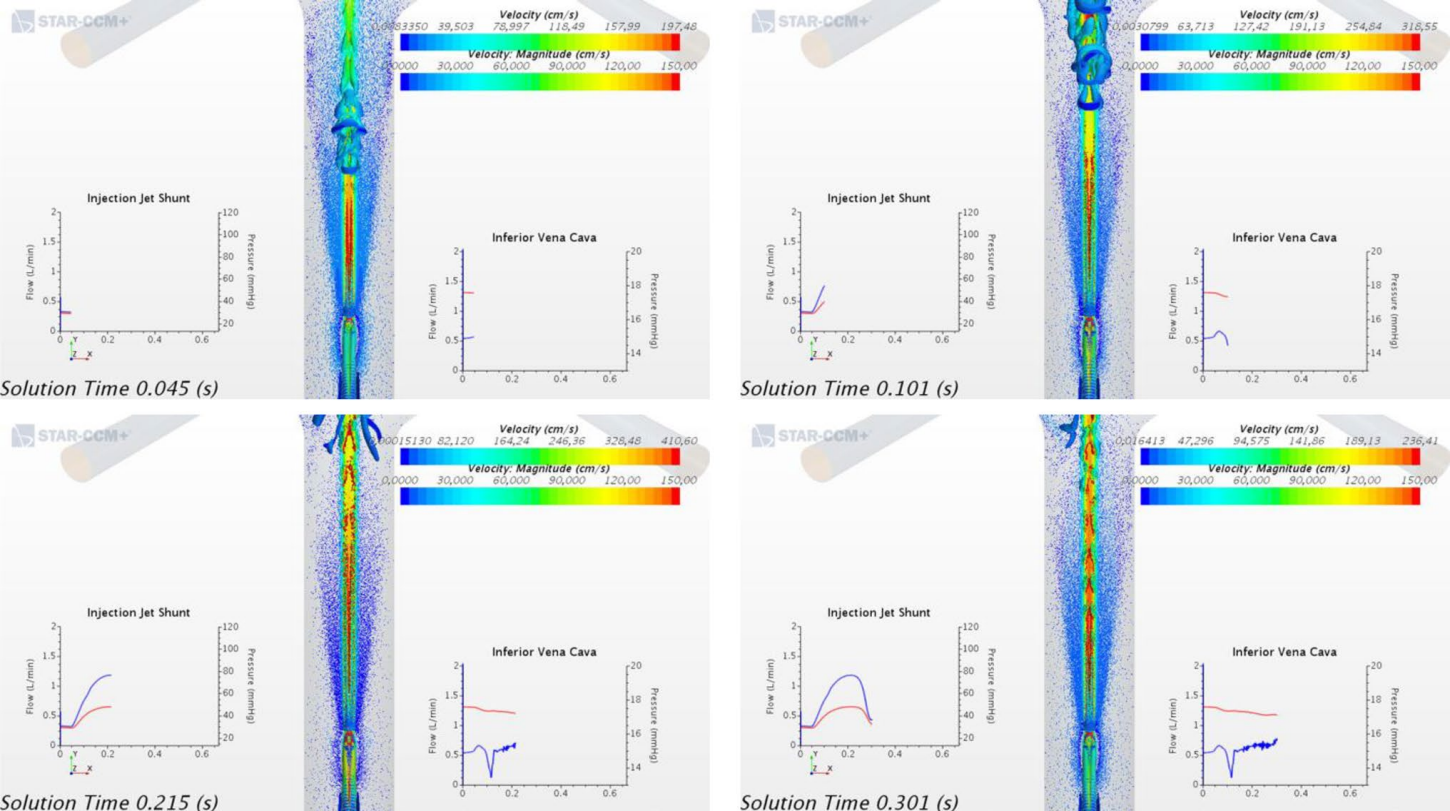
Contour velocity plot of vortex iso-structures for a 2 mm circular IJS nozzle, 7 cm IJS-TCPC distance and 7 mm fenestration across a single heart cycle. The x–y plots display pressure (blue) and volume flow rate (red) sampled in the IJS shunt and the IVC. Displayed are the flow velocity (“Velocity”) and coherent structure velocity (“Velocity: Magnitude”).

Figures 4 and 5 displays of the flow field between the onset of systole and mid-diastole (lasting about 0.3 s), as the jet transitions from laminar to turbulent and back from turbulent to laminar with flow field velocities are varying most significantly. We focus on that portion of the cardiac cycle, the first 0.3 s of the full 0.65 s of the cardiac cycle corresponding to 90 BPM, where the IJS is active and interacting with the caval ambient flow.

Figure 4 displays the velocity magnitude contour plots focused in the Fontan conduit region to highlight jet co-flow turbulent interactions along the major and minor axis. This figure offers insight on the significant velocity difference between the jet and the surrounding flow as well as the transient nature of the jet driven by systemic pulsatile flow. In late diastole the jet speed is ~ 200 cm/s, reaching nearly 420 cm/s in peak systole. While the background flow retains rather low speeds of ~ 20 to 70 cm/s across a cycle. Evidence of entrainment can be observed for a variety of qualitative outcomes. As the jet moves downstream, its core gets dissipated about 7–12 IJS diameters downstream while the cone grows indicating recruitment of surrounding IVC flow and momentum transfer. IVC flow recruitment implies a significant inward radial velocity component for the surrounding flow seen here in the converging flow around the jet volume. Lastly, a key mechanism of entrainment is shearing resulting in eddies and vortices, especially evident in the transition from late diastole to mid-systole. The shunt geometry, as shown in Fig. 1, has the IJS implemented with a notable curvature expected to induce secondary flows or Dean flow along the minor axis. The minor axis sequence in Fig. 4 confirms this outcome. The velocity profile shows the typical skew induced by Dean flow resulting in an asymmetric jet cone growth downstream. This has a mild entrainment effect given the jet core in no longer centered, potentially causing flow instability. Along the major axis, symmetry is maintained. While averaging the IVC pressure waveform reveals the significant pressure drop achieved thus far, a closer look at the IVC pressure wave form in Fig. 4 shows a remarkable *transient* pressure drop to 13.441 mmHg. This coincides with the onset of systole resulting in a jet burst that generates vortical structures quickly convected downstream suddenly enhancing entrainment. It must be emphasized however that from a clinical perspective the mean IVC pressure drop is of much greater relevance than any transient effect. This behavior, however, may ultimately guide one to additional IJS configurations, such as multiple higher velocity jets that could be “additive” to the injection jet effect (as long as CO/Qs < 1.5).

The importance of vortical structures as these relate to the mechanism of entrainment was noted in the analysis of Fig. 4. Since the jet is powered by systemic pressures, the ejected flow is highly pulsatile. Hence the induced structures also form periodically and are convected in a pulsatile manner. Figure 5 displays vortices based on the lambda-2 criterion to track coherent iso-structures. Preliminary vorticity quantification for the cut-off values are used. Here, the sequence shows three distinct types of vortices: ring vortices, hairpin vortices, and longitudinal (helicoidal) vortices. Ring vortices emerge from the shear layer interface between the jet and the surrounding flow. In order to inject flow along the Fontan conduit centerline, the IJS protrudes into the incoming IVC flow, this configuration can be loosely compared to a blunt body in crossflow which typically induces hairpin vortices. Longitudinal vortices arise from the induced Dean flow and shed from the shunt wall exposed to IVC flow. Ring and hairpin vortices are the main drivers of entrainment as they present with larger and more distributed radial velocity components along the azimuthal direction. Longitudinal vortices can have very localized radial velocity components however their contribution to entrainment emerges from their interaction with ring vortices. These vortices can induce instability in ring vortices causing the toroid to warp symmetrically and then potentially to break up in hairpin like structures. The degree of symmetric warping is dictated by the symmetry of the secondary Dean flow generated in the IJS. As these toroids move downstream, their asymmetry grows, generating uneven entrainment in the azimuthal direction.

Figure 5 offers additional insight on the bulk velocity of these structures. The rate at which large vortices are transported downstream can affect IVC pressure. In the extreme case that these structures were to be nearly static, it could potentially indicate or lead to a significant degree of blockage. In this figure, the bulk velocity of these structure varies depending on their location in the lumen and across the heart cycle. Smaller eddies found near the jet core move comparatively faster (speeds in the excess of 150 cm/s) than larger vortex rings spanning the lumen (< 50 cm/s). Vortices transported at peak systole will experience higher convective velocities than vortices in diastole.

## Discussion

This paper presents a comprehensive study that reveals a significant IVC pressure drop by means of an injection jet shunt in computational models of the failing Fontan. First, it is demonstrated that merely increasing fenestration size, which intuitively would lead to a clinically relevant IVC pressure reduction, also reduces pulmonary flow and results in a right-to-left shunt and unacceptable systemic oxygen content. An IJS operating in tandem with the fenestration, however, can induce entrainment, momentum transfer, reduction in IVC pressure and (in principle) acceptable systemic oxygen content.

The implementation of an oxygen tracking scheme to monitor saturations, an LES turbulence model to accurately simulate the pulsatile co-flowing jet, and the use of two-phase flow modeling to quantify the systemic blood volume fraction absorbed by the fenestration, allowed the exploration of several geometrical parameters to identify a preliminary optimal configuration. These parameters include anatomic variants of the Fontan connection (e.g., 2 Y-grafts), the placement and size of the IJS shunt, and the location and diameter of the fenestration. In group 1, the parametric search found that the IJS nozzle diameter is more impactful than the IJS-cavopulmonary connection distance in altering Fontan hemodynamics. A smaller IJS nozzle diameter yielded greater jet velocity and entrainment and had the collateral benefit of limiting CO/Q_s_. In group 2, increase in fenestration size was found to “offload” the accumulation of volume proximal to the lung impedance due to the IJS mass flow. This resulted in decreased IVC pressure and somewhat decreased Qp/Qs. Finally, a successful model with a 2 mm IJS located 7 cm from the cavopulmonary connection and an 8 mm fenestration, resulted in a 3.2 mmHg IVC pressure drop with systemic oxygen saturation around 80%. Two additional IJS-fenestrated Fontan configurations that provide surgically feasible alternatives with similar levels of IVC pressure drop and systemic oxygen concentration were also presented. As shown, IVC pressure can be significantly reduced in all of these models, but systemic oxygen saturation is not always acceptable. It is clear that further improvement in oxygen delivery can be achieved by increasing the fraction of total fenestration flow that originates from the IJS. The optimal model may be one in which the fenestration diameter is somewhat smaller than 7–8 mm, and that the IJS jet diameter matches the fenestration diameter at the location of the fenestration. In this way, the jet flow “displaces” more of the ambient (de-oxygenated) flow away from the fenestration, allowing jet flow to be the predominant mass that traverses the fenestration. This may be achieved by further optimization of the geometric parameters of the model, and will be a principal thrust of our future work.

There are several limitations to the present study. First is the rigid-wall assumption which obviates the need for considering fluid–structure interaction. This simplification is reasonable as the Fontan conduit is made of a non-compliant Gore-Tex graft and the pulse pressure in the Fontan circulation is small^69,70^. Second, the complexity of our models require validation^71^. While the results are currently being validated with in-vitro simulations, it is preferable to also validate with independent computational modeling. Third, the current model does not account for collateral flow, which is not uncommon in Fontan patients and which, together with the IJS shunt could result in excessive cardiac volume load (CO/Qs > 1.5). This problem could be addressed, however, with catheter intervention and collateral occlusion. Fourth, in this study a 2 mm nozzle was identified the most beneficial configuration for the IJS, such a small shunt could potentially be thrombogenic. Fontan post-operative management, however, typically includes pharmacologic anticoagulation which may reduce the risk of thrombosis, making the shunt size less problematic. Fifth, as the patient grows, systemic and pulmonary flow increase thereby altering the co-flow dynamics with the injection jet. Thus, if our model is to be applied to longer term therapy, an increase in IJS mass flow might be required. We speculate that this could be achieved by catheter intervention to enlarge the shunt.

While we have described the potential therapeutic benefits of this proposed mechanism, we have not defined who would benefit the most, and when the procedure should be performed. As we have described in our prior work, the mechanism specifically addresses lowering of the Fontan pressure (P_*fontan*_). Clearly, in patients will intrinsically failing myocardium, atrioventricular valve insufficiency, or significantly elevated PVR, our mechanism is unlikely to confer effective palliation. The decision of whether to perform the procedure at the time of the initial Fontan operation, or later as indicated is not yet clear and beyond the purview of the present work.

In our model, pulmonary vascular resistance (PVR) was held constant. There is some evidence that PVR may decrease with increased pulmonary blood flow and some degree of pulsatility^72,73^. If so, then the IJS may have this additional collateral benefit.

In conclusion, a reduction of Fontan pressure (IVC pressure) using an injection jet powered by the native single ventricle is demonstrated in this detailed computational study. Further work will enable optimization of the anatomic and physiological requirements of this method. Given the substantial challenges of clinically implementing externally powered mechanical pumps, the more straightforward mechanism presented here may be a more rapidly applicable, albeit palliative method to treat the increasing population of Fontan patients at risk for failing.

## Supplementary Information

Supplementary Information.

## Data Availability

All data generated or analyzed during this study are included in this published article.

## Abbreviations

2Y: Double Y-graft
BC: Boundary conditions
BDF: Backward finite difference
BSA: Body surface area
CFD: Computational fluid dynamics
CO: Cardiac output
IJS: Injection jet shunt
IVC: Inferior vena cava
LES: Large eddy simulation
LPA: Left pulmonary artery
LPM: Lumped parameter model
PVR: Pulmonary vascular resistance
Qp: Pulmonary flow
Qs: Systemic flow
RPA: Right pulmonary artery
SV: Single ventricle
SVC: Superior vena cava
TCPC: Total cavopulmonary connection
WALE: Wall adapted local eddy viscosity
