# Supplementary material for: Parametric investigation of an injection-jet self-powered Fontan circulation
Source: Sci Rep. 2022 Feb 9;12:2161. doi: 10.1038/s41598-022-05985-3 (PMC8828777; doi:10.1038/s41598-022-05985-3)
Supplement: Supplementary file 1 — Supplementary Information. [file 41598_2022_5985_MOESM1_ESM.docx]

**Title**: Parametric Investigation of an Injection-Jet Self-Powered Fontan Circulation

**Authors**:

Ray Prather, PhD ^1,2,3^

Arka Das, PhD ^2^

Michael Farias, MD, MBA ^3^

Eduardo Divo, PhD ^2^

Alain Kassab, PhD ^1^

William DeCampli, MD, PhD ^3,4^


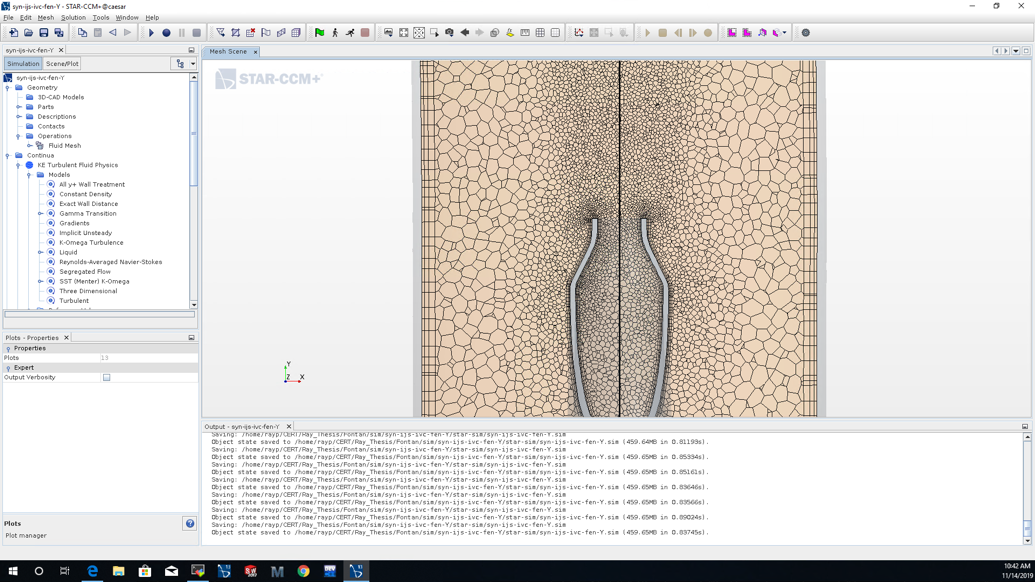

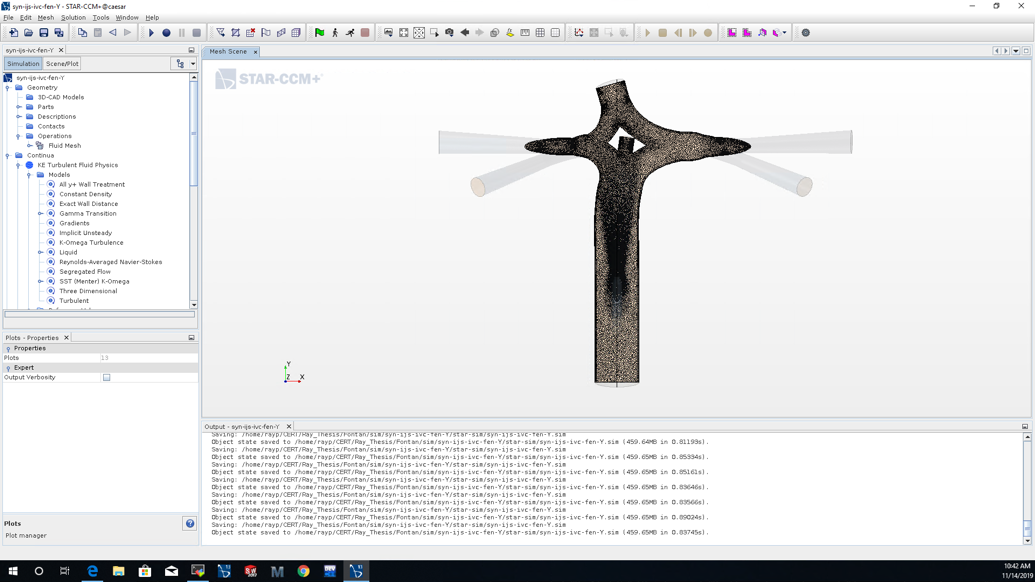

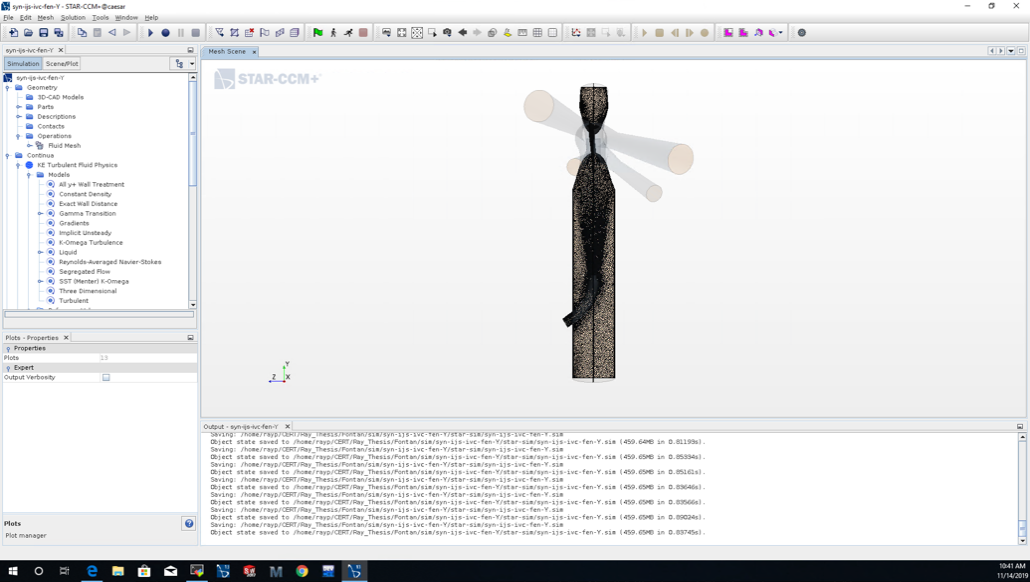


A

B

Supplementary Figure S1 - Volume mesh refinement based on shunt surface and shunt mouth wake ((A) front and side full domain view and (B) close-up to IJS outlet).


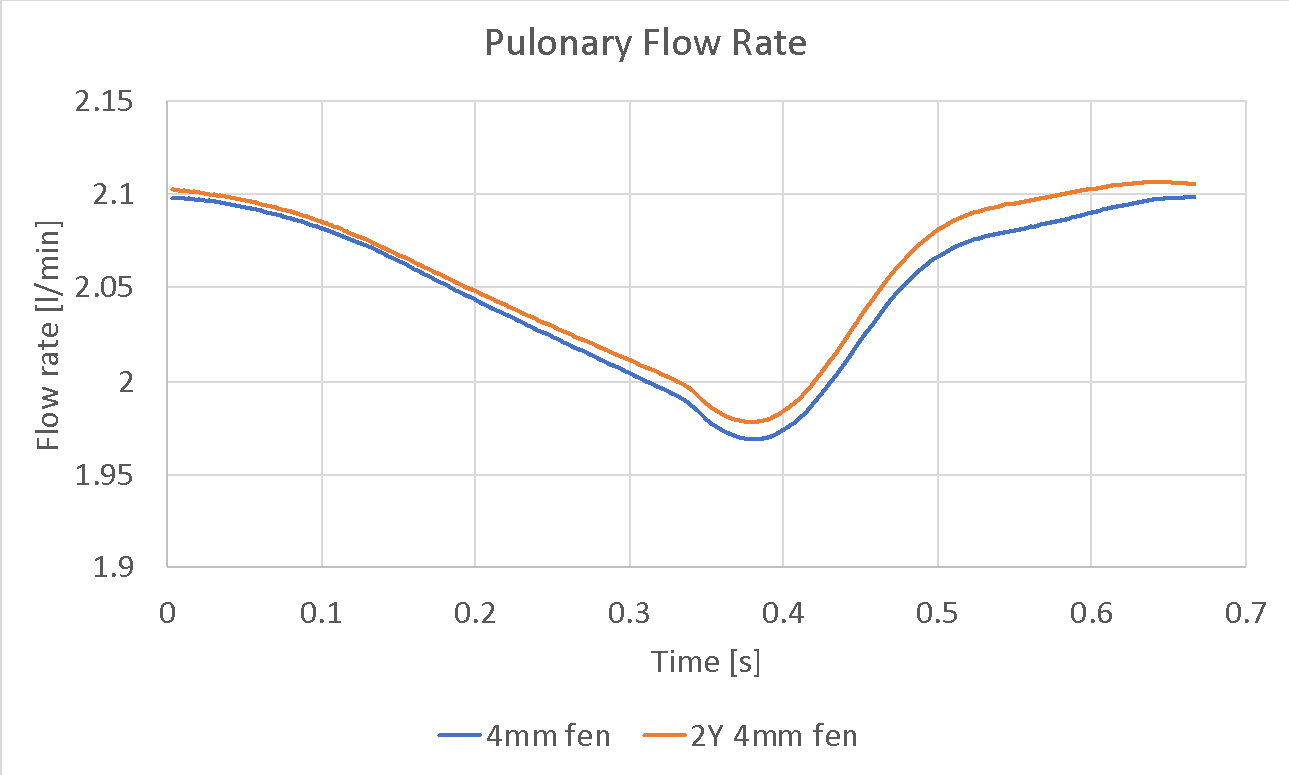

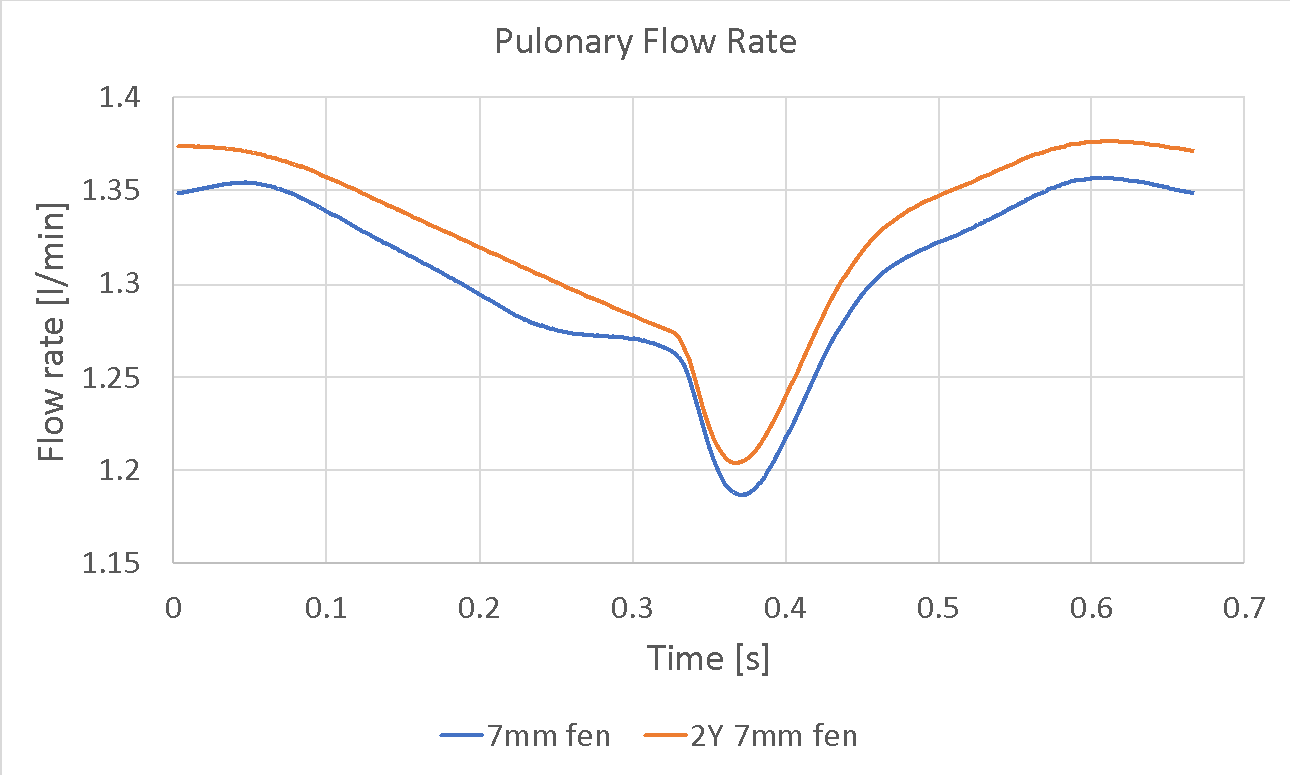


A

B

Supplementary Figure S2 - Qp for a set of fenestrated Fontan models with a standard (baseline) connection and a double Y-graft anatomic variant for (A) a 4mm fenestration and (B) a 7mm fenestration.
